# Quantification of biomaterial dispersion during otologic procedures and role of barrier drapes in Covid 2019 era – a laboratory model

**DOI:** 10.1017/S002221512000239X

**Published:** 2020-11-04

**Authors:** P K Lokesh, S Chowdhary, S A Pol, M Rajeswari, S K Saxena, A Alexander

**Affiliations:** 1 Department of ENT, Jawaharlal Institute of Postgraduate Medical Education and Research (‘JIPMER’), Puducherry, India; 2 Department of Biostatistics, Jawaharlal Institute of Postgraduate Medical Education and Research (‘JIPMER’), Puducherry, India

**Keywords:** Temporal Bone, Surgery, Aerosols, Surgical Drapes

## Abstract

**Background:**

Aerosol generation during temporal bone surgery caries the risk of viral transmission. Steps to mitigate this problem are of particular importance during the coronavirus disease 2019 pandemic.

**Objective:**

To quantify the effect of barrier draping on particulate material dispersion during temporal bone surgery.

**Methods:**

The study involved a cadaveric model in a simulated operating theatre environment. Particle density and particle count for particles sized 1–10 μ were measured in a simulated operating theatre environment while drilling on a cadaveric temporal bone. The effect of barrier draping to decrease dispersion was recorded and analysed.

**Results:**

Barrier draping decreased counts of particles smaller than 5 μ by a factor of 80 in the operating theatre environment. Both particle density and particle count showed a statistically significant reduction with barrier draping (*p* = 0.027).

**Conclusion:**

Simple barrier drapes were effective in decreasing particle density and particle count in the operating theatre model and can prevent infection in operating theatre personnel.

## Introduction

The severe acute respiratory syndrome coronavirus-2 (SARS-CoV-2) caused the outbreak of coronavirus disease 2019 (Covid-19) that started in Wuhan, China, in December 2019. The World Health Organization (WHO) declared Covid-19 a pandemic in March 2020. So far, more than 22 million people have been infected worldwide, with more than half a million deaths.^1^

The virus spreads via aerosols, and the nasal and nasopharyngeal regions are associated with high viral loads, thereby putting healthcare professionals, particularly otolaryngologists, at risk of contracting the infection.^2^ Multiple reports have quoted the high risk to practising otolaryngologists performing various office-based and surgical procedures.^3–7^ Existing literature shows that the use of high-speed drills generates aerosols that have the potential for dissemination of bacteria,^8^ viruses^9^ and prion particles^10^ in the operating theatre.

No studies to date have quantified the dispersion of biomaterial or particulate matter dynamically inside a simulated conventional operating theatre at the time of bone drilling as part of otological and neurotological procedures. Our study attempted to quantify the particulate matter dispersion during these procedures. It also aimed to determine the efficiency of preventive measures recently proposed to minimise the dispersion of biomaterial in otological procedures.

## Materials and methods

### Experimental setup

A temporal bone dissection laboratory facility in a tertiary care teaching hospital was used in the current study; this was equipped with air exchangers operating at six cycles (air changes) per hour. All the non-essential equipment was taken out of the laboratory. The laboratory was cleaned and wet-mopped to remove fine dust before performing the study, to simulate an operating theatre environment.

Temporal bone drilling was carried out with a high-speed drill, using various sizes of cutting burrs, at 35 000 revolutions per minute with a Zeiss Opmi microscope (Carl Zeiss Meditec, Jena, Germany). Drilling was conducted for 3 consecutive days on three human temporal bones preserved in formalin.

Particle density and particle concentration for three different particle sizes (i.e. 1 μ, 2.5 μ and 10 μ) was measured before performing the bone drilling (baseline) with a Prana Air PM2.5 pocket monitor (Purelogic Labs, Schezhen, China). This pocket monitor takes in 0.1 litre per second of air and uses a laser sensor to determine the particle density based on the scattering of light. It has a resolution of 1 μg/m^3^, with an accuracy of 0–150 μg/m^3^ ± 10 per cent.

**Fig. 1.**
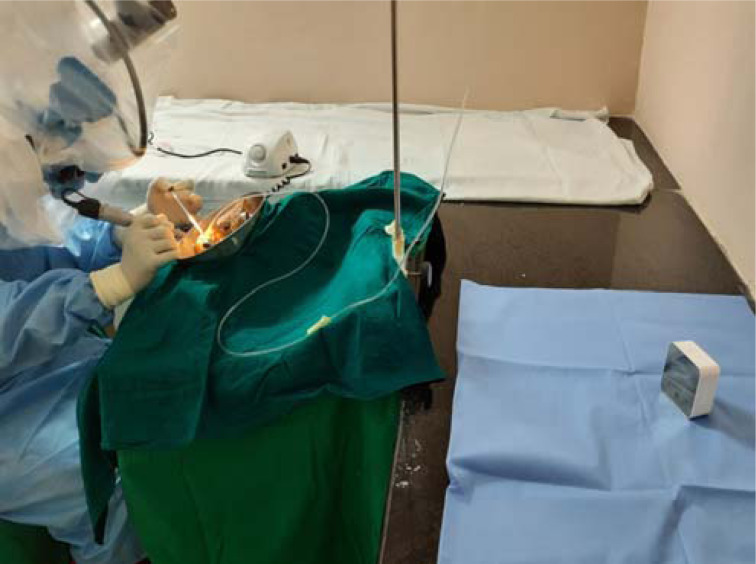
Pocket monitor placed 100 cm from the mastoid bone in the setup of drilling without drapes.

**Fig. 2.**
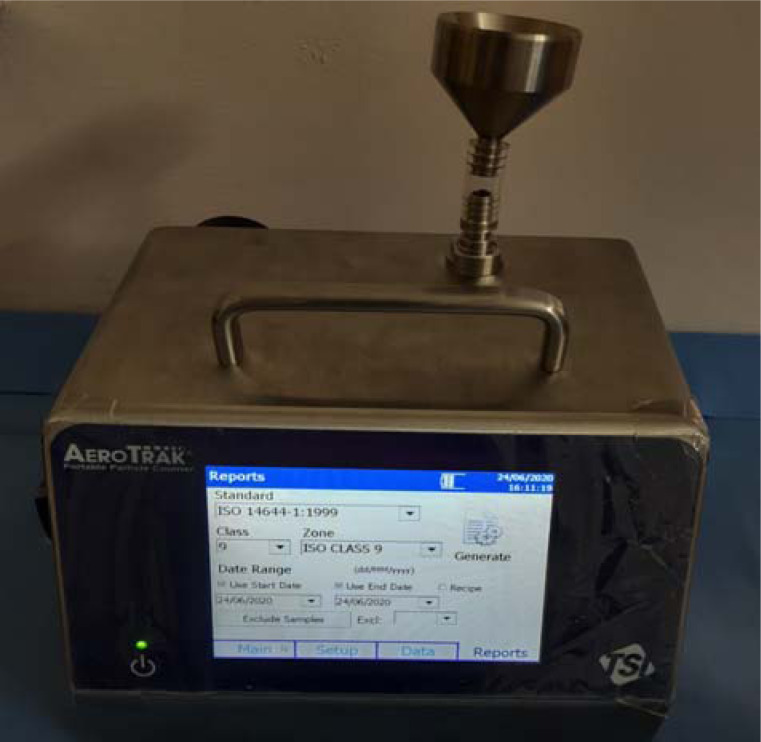
Portable particle counter placed at a far distance from the site of drilling inside the operating theatre.

The temporal bones were denuded of soft tissue. Cortical mastoidectomy was performed with the high-speed drill. Particle density was then measured by placing the pocket monitor at 50 cm and 100 cm distance from the site of drilling during the procedure (Figure 1). Particle count (number of particles per cubic meter) for different particle sizes (i.e. 0.5 μ, 1 μ and 5 μ) was measured before and after cortical mastoidectomy with a TSI AeroTrak® portable particle counter (model 9500) (Figure 2), which was placed within the laboratory but distant to the site of drilling. International Organization for Standardization ISO 14644 class 9 cleanroom standards were set in the TSI AeroTrak portable counter to assess the particle count.

Measurements of particle density and particle count were taken initially in the setup with barrier drapes and then repeated without barrier drapes. An ample amount of time was maintained between the two setups so that the dispersed particles in one setting would not contaminate the other.

### Barrier drapes

**Fig. 3.**
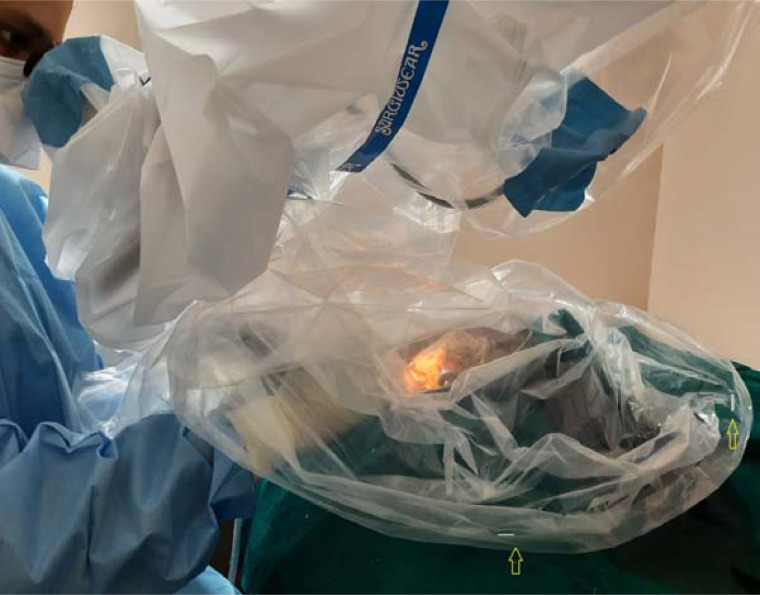
Simple barrier drapes fixed to the lens mount of the microscope and stapled (yellow arrows) to the drapes over the patient cart. The surgeon's hands and instruments were introduced through the gaps between the staples on the surgeon's side.

A transparent plastic drape measuring 200 × 100 cm was used. A circular hole of 6 cm diameter was made at the centre of the drape and affixed to the lens mount of the microscope with adhesive tape. The drape was aligned so that the objective lens was not obstructed. The barrier drape was loosely rolled under and fixed all around the drilling site 40 cm away, at three points, with staples, to the right, left and opposite the surgeon (Figure 3). The surgeon's arms and instruments were passed under the drapes on the surgeon's side. Following each day of drilling, the drape was carefully removed and disposed of, to avoid spillage of particulate matter inside the laboratory.

### Statistical analysis

The particle analysis concerned two parameters: particle density (recorded in micrograms per cubic meter) for different sizes of particles (1 μ, 2.5 μ and 10 μ) using the Prana Air equipment; and particle count (the number of particles per cubic meter) for different sizes of particles (0.5 μ, 1 μ and 10 μ) using the TSI Aero Trak® equipment. The latest version of SPSS® software, version 19.0 (19.0.0.2), was used for statistical analysis.

### Particle density analysis

Particle density for different sizes (1 μ, 2.5 μ and 10 μ) of particulate matter was recorded, and descriptive statistics, including mean, median and interquartile range, were calculated. Statistical significance between particle densities recorded at 50 cm from the drilling site while drilling with and without drapes was determined using the Mann–Whitney U test. Similarly, the difference between densities at 100 cm from the drilling site while drilling with and without drapes was calculated. Kruskal–Wallis test was used to test for statistically significant differences between medians for various test conditions (baseline, with and without drapes at 50 cm and at 100 cm).

### Particle count analysis

Particle count per cubic meter was measured for different sizes (0.5 μ, 1 μ and 5 μ) of particle matter, and descriptive statistics, including mean, median, and interquartile range, were determined. Statistical significance of the difference between particle counts during drilling with and without drapes was measured using the Mann–Whitney U test. Similarly, the Kruskal–Wallis test was used to test for statistically significant differences between the various test conditions (baseline, with drapes and without drapes).

## Results

Biomaterial or particle matter dispersion was measured as particulate concentration or density, and particle count. Particle density was measured at baseline, with drapes at 50 cm and 100 cm from the site of drilling (approximately at the external auditory canal), and without drapes at 50 cm and 100 cm from the site of drilling. Particle count was measured at baseline, with and without drapes.

### Particle density

**Fig. 4.**
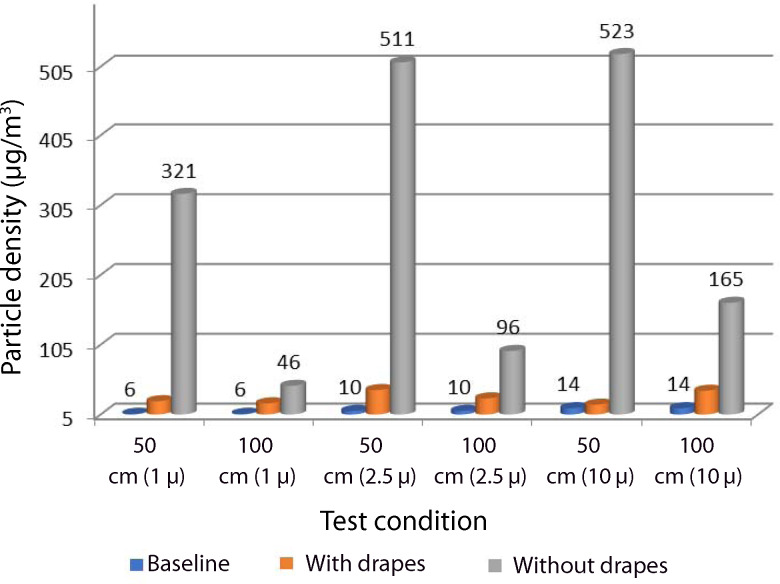
Particle densities under the various test conditions (distance from site of drilling (for each particle size)). The chart shows the significant rise in the median particle densities during drilling without drapes when compared to the other two test conditions, for all three sizes of particulate matter.

The median particle density values for different particle sizes (1 μ, 2.5 μ and 10 μ) in the different test conditions are depicted in Figure 4.

Particle density for all three particle sizes (1 μ, 2.5 μ and 10 μ) were compared at the two different distances from the site of drilling, namely with and without drapes at 50 cm, and with and without drapes at 100 cm. The number of increases in particle density from baseline in the different test conditions, and the medians and interquartile ranges, are illustrated in Table 1.

**Table 1.** Median particle densities in different test conditions

Particle size	Test condition	Median (IQR) particle density (μg/m^3^)	Number of times rise in particle density from baseline	*P*-value (Kruskal–Wallis test)
1 μ	With drapes at 50 cm from EAC	24 (23–25)	4	0.027
	Without drapes at 50 cm from EAC	321 (315.5–337)	53	
	With drapes at 100 cm from EAC	21 (20.5–22.5)	5	
	Without drapes at 100 cm from EAC	46 (45–46.5)	11	
2.5 μ	With drapes at 50 cm from EAC	40 (40–42)	4	
	Without drapes at 50 cm from EAC	511 (505–518.5)	51	
	With drapes at 100 cm from EAC	28 (27–28.5)	3	
	Without drapes at 100 cm from EAC	96 (94.5–96.5)	10	
10 μ	With drapes at 50 cm from EAC	19 (18.5–20)	2	
	Without drapes at 50 cm from EAC	523 (468.5–527)	37	
	With drapes at 100 cm from EAC	39 (38.5–40)	3	
	Without drapes at 100 cm from EAC	165 (164.5–168)	12	

IQR = interquartile range; EAC = external auditory canal

The particle density of 2.5 μ and 10 μ sized particles was higher as compared to 1 μ sized particles when measured without drapes at 50 cm. The median particle density was at least five times higher without drapes at 50 cm from the drilling site compared to without drapes at 100 cm (511 μg/m^3^ and 96 μg/m^3^ for 2.5 μ particles, respectively). However, there was no statistically significant difference between the densities in terms of distance from the external auditory canal (*p* > 0.05, Mann–Whitney U test). Comparison of the particle densities for all three particle sizes with and without drapes (i.e. with and without drapes at 50 cm, and with and without drapes at 100 cm) showed a statistical difference (*p* = 0.046, Mann–Whitney U test). There was a statistically significant difference in the particle densities when comparing the test conditions (baseline, with or without drapes at 50 cm, and with or without drapes at 100 cm) for all the sizes of particles using the Kruskal–Wallis test (*p* = 0.027).

### Particle count

**Fig. 5.**
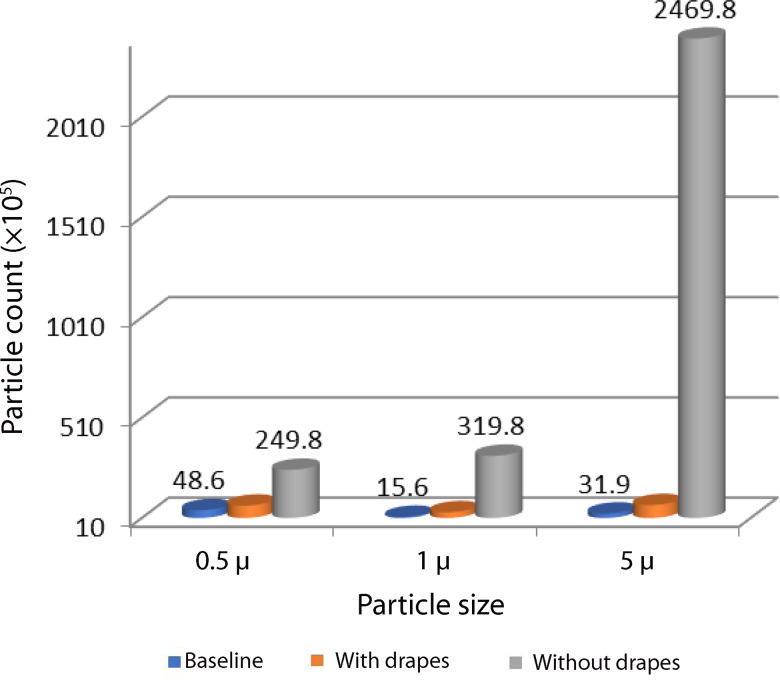
Particle counts under the various test conditions. A significant rise in the particle count is noted during drilling without drapes.

Median particle counts for different particle sizes (0.5 μ, 1 μ and 5 μ) are illustrated in Figure 5.

The number of times rise in the particle count from baseline, and the medians and interquartile ranges, for different particle sizes, are depicted in Table 2.

**Table 2.** Median particle count in different test conditions

Particle size	Test condition	Median (IQR) particle count (×10^5^)	Number of times rise in particle count from baseline	*P*-value (Kruskal–Wallis test)
0.5 μ	With drapes	71.4 (70.4–72.5)	1.5	0.027
	Without drapes	249.8 (237.7–259.4)	5	
1 μ	With drapes	38.4 (37.4–39.5)	2.5	
	Without drapes	319.8 (305.4–340.5)	21	
5 μ	With drapes	74.1 (71.5–75.3)	2	
	Without drapes	2469.8 (2166.5–2716.7)	80	

IQR = interquartile range; EAC = external auditory canal

The counts for 5 μ sized particles were 80-fold higher during drilling without drapes as compared to particles sized 0.5 μ and 1 μ. Similar to particle densities, the particle counts showed a statistically significant difference between the two test conditions (with drapes and without drapes, *p* = 0.05; Mann–Whitney U test). There was also a statistically significant difference in the particle counts between baseline, with drapes and without drapes when multiple comparisons were analysed with the Kruskal–Wallis test (*p* = 0.027).

## Discussion

Coronavirus disease 2019 has become a serious threat, not only to the general population but to healthcare workers, with recorded higher mortality rates. Otorhinolaryngologists have a higher risk of contracting the virus as they perform procedures in nasal, oral and oropharyngeal regions, which are areas identified as having a high viral load. There is an anecdotal report of up to 14 healthcare workers in a single operating theatre contracting the infection after an endoscopic pituitary surgical procedure in Wuhan.^5^

There is no clear consensus about otological surgical procedures as aerosol-generating procedures. However, owing to the communication of the middle ear with high viral laden areas such as the nasopharynx, otological and skull base procedures are theoretically riskier than they would be for operating personnel. There are no published data regarding the quantification of biomaterial or particulate matter dispersed during otological procedures in the setting of an operating theatre. This study attempted to quantify particulate matter dispersion in a simulated operating theatre setting. It also aimed to assess the efficiency of mitigation procedures such as the use of otological barrier drapes recently adopted for performing emergency otological procedures.

A cortical mastoidectomy is a fundamental surgical step in any otological and neurotological procedure, elective or otherwise. Hence, biomaterial or particulate matter dispersion measurements were made while performing this procedure. The procedure was carried out on a formalin preserved cadaveric temporal bone specimen using a high-powered drill at various speeds, operated by a right-handed surgeon.

The generation of aerosol sized particles when using a high-powered drill in an operating theatre setting has been discussed previously.^9^ The WHO defines a droplet particle as a particle sized 5–10 μ and a droplet nucleus if smaller than 5 μ.^9^ In the context of Covid-19, the WHO has declared that the virus can spread by airborne transmission when performing procedures or support treatments that generate aerosols.^10^ Using these definitions of droplet particle and nuclei in our experimental setup, particle density for particle sizes 1 μ, 2.5 μ and 10 μ, and particle count per cubic meter for particle sizes 0.5 μ, 1 μ and 5 μ, were measured while performing cortical mastoidectomy in a closed simulated operating theatre environment in two different settings: with barrier drapes and without barrier drapes.

There have been reports of middle-ear and inner-ear fluids laden with respiratory viruses during otitis media and upper respiratory infections.^11,12^ While there is anecdotal evidence that plumes and fine bone dust may be found on the drapes and surgeons’ gowns after mastoid drilling, there is presently no available literature to quantify particulate dispersion. There is very little available literature on how to quantify this particulate dispersion.^13^ Particles sized between 1 μ and 10 μ are of importance in this Covid-19 era. We analysed the particulate dispersion according to particle density and particle count measured using the Prana Air PM2.5 pocket monitor and TSI AeroTrak portable particle counter (model 9500), respectively.

In the present study, the particle density for particles sized 1 μ, 2.5 μ and 10 μ was significantly higher at a distance of 50 cm from the external auditory canal while drilling without drapes as compared to baseline densities. Though the median particle density was five-fold higher with drapes at 50 cm compared to with drapes at 100 cm for both 2.5 μ and 10 μ particles, there was no statistically significant difference between the densities while drilling without drapes at 50 cm and 100 cm from the mastoid bone.

Chen *et al*. performed a semi-quantitative study to measure the surface particle density using a fluorescent dye and an ultraviolet light source, and found that particle density was greater at 30 cm from the mastoid bone.^13^ However, because of image resolution limitations, they could not evaluate particles smaller than 100 μ. The authors noted that the particle densities were lower when working under the drapes (the OtoTent®). The present study found that use of a barrier drape significantly reduced the particle density for all sizes of particles, both at 50 cm and 100 cm from the mastoid bone.

At present, there is no consensus regarding the best practises for preventing viral transmission inside operating theatres. Various mitigation strategies have been proposed for different procedures such as intubation and extubation,^14^ tracheostomies and endoscopic skull base procedures.^4^ Simple barrier drapes have been recommended to reduce the particle densities outside the drapes during mastoidectomy.^13^ The major drawbacks with the simple barrier drapes included difficulty in passing instruments, escape of droplet nuclei sized particles from the gaps around the drapes, and accumulation of bone dust on the drapes which could impact visualisation in the later part of the surgery. The droplet particle sizes that are of concern in terms of viral transmission (smaller than 10 μ) were not quantitatively evaluated in that study.^13^

In our study, particle count for particles sized 0.5 μ, 1 μ and 5 μ, which are of the droplet range, were measured during drilling, with and without drapes. Compared to the baseline particle count, which was measured before performing the drilling, the median particle count for 5 μ sized particles was elevated 80 times over while drilling without the barrier drapes, even at a far distance inside the operating theatre (31.9 × 10^5^ per cubic meter at baseline, compared to 2469.8 ×10^5^ per cubic meter when drilling without drapes), which could pose a risk of infection to other staff. The use of barrier drapes significantly reduced particle counts for 5 μ sized particles when compared to particles sized 0.5 μ and 1 μ (*p* = 0.046, Mann–Whitney U test). Hence, simple barrier drapes alone, without any further modification, can decrease biomaterial or particle density and particle count to some extent.

Modified barrier drapes with a second outer tent (the OtoTent 2) and the use of a second suction close to the drilling area were shown to reduce particle density in a recent study.^15^ However, static methods such as optical particle sizers were used, and particle counts in the operating theatre far from the site of drilling were not evaluated; only particle density and particle count very close to the mastoid bone (30 cm) were measured.

- Temporal bone surgery generates aerosols of particles in the droplet range that carry the risk of viral transmission
- These particles were measured in significant densities at 50 cm from the drilling site
- Particles smaller than 5 μ were found in substantial amounts throughout the operating theatre
- The use of barrier drapes reduces the dispersion of particles smaller than 5 μ by a factor of 80
- Otological barrier draping is vital to prevent infection in operating theatre staff, especially during the current coronavirus disease 2019 pandemic

The relevance of aerosolised biomaterial in the spread of highly contagious infections like Covid-19 during mastoidectomy using a high-speed drill is still unclear. Norris *et al*.^16^ found that the average particle count during cortical mastoidectomy was 1.89 mg/m^3^. They concluded that calculated particulate exposure concentrations for total soluble particulate matter did not exceed Occupational Health and Safety Administration requirements for respirator use. In our study, the median particle densities for 1 μ, 2.5 μ and 10 μ particles immediately following drilling were 0.18 mg/m^3^, 0.33 mg/m^3^ and 0.34 mg/m^3^, respectively, which is also within the safety limits as recommended by the Occupational Health and Safety Administration. However, whether this particle density of infected material can cause transmission of Covid-19 is not known.

As there is insufficient evidence regarding the exact quantity of aerosolised biomaterial which poses a transmission risk, and because of various lacunae in the mitigation techniques, we recommend withholding all non-emergency otological procedures until polymerase chain reaction antigen testing is twice negative. We also strongly recommend the use of personal protective equipment for all emergency otological procedures, in the form of impervious gowns with head coverings, wrap-around eye goggles, and N95 masks (which filter particles smaller than 0.3 μ) for all operating theatre personnel, as the present study demonstrated a high concentration of particles smaller than 5 μ in the operating theatre.^17^ The use of high efficiency particulate air (‘HEPA’) filters, negative pressure ventilation and dilution ventilation systems in the operating theatres would help to decrease the concentrations of transmissible biomaterials.^18,19^

### Study strengths

Biomaterial or particle dispersion around the site of drilling (50 cm and 100 cm distance from the site) was quantified, knowledge of which is important for the safety of surgeons and assisting personnel. Particle count was measured inside the operating theatre, but at a distance from the site of drilling, which helps in assessing the risk to other staff. As simple barrier drapes decrease particle density and particle count for particles larger than 5 μ, their use would be a basic recommendation for all otological procedures. Modifications in the barrier drapes, such as the use of a second outer drape and a second suction close to the site of drilling, are not without benefit, but result in a longer draping time.

### Study limitations

The temporal bone laboratory cannot replicate operating theatre conditions. The cadaveric bone models differ from a live bone, which may have a bearing on results. The effects of various drill speeds, drilling durations and amounts of irrigation were not tested in our study. Further studies are required to quantify the virus in the aerosolised biomaterial generated from mastoid drilling and its neutralisation strategies.

## Conclusion

Particle densities in the droplet range were measured close to and away from the site of drilling in two setups (with barrier drapes and without drapes). The densities of 2.5 μ and 10 μ sized particles were significantly higher around the operating site during and immediately after drilling (at 50 cm circumference) without barrier drapes. The particle count for particles smaller than 5 μ was noted to be significantly elevated in the operating theatre, even at a distance from the site of drilling (without barrier drapes), thus posing a risk of infection to other personnel in the operating theatre. Both the particle densities and particle counts were significantly reduced with the use of simple barrier drapes. Various scientific papers,^13,15,20^ including the present study, support the use of various barrier drapes for otological surgical procedures, but they must be used in conjunction with adequate personal protective equipment.
